# A Word to the Wise

**Published:** 1889-11

**Authors:** 


### A WORD TO THE WISE.

The most prominent characteristic of the present age, from a medical point of view, is the wonderful development of the art of surgery. No previous period can compare with it in the progress which has been made in this department of medicine. Regions of the body, which a few years ago were deemed inaccessible to the knife, are now fearlessly laid open, diseased portions removed and new growths extirpated with results which fully justify the boldness of such procedures. The abdominal cavity has become a play-ground for ambitious surgeons; the brain and the spinal cord have become legitimate fields for surgical work, and even the pericardium has been punctured with impunity.

The brilliancy and *eclat* of major surgical operations give to them a peculiar fascination, especially to the younger portion of the profession. The removal of an abdominal tumor, the location and puncture of an abscess of the brain, the successful drainage and cure of a suppurating synovial membrane charm the mind of the beholder and give to the operator fame and distinction, such as are not acquired in any other branch of medical practice. An ambition to excel in such lines of work is a worthy and laudable one, and we have no sympathy with the spirit which would deplore the prevailing tendency in this direction. Countless lives have been saved and immeasurable suffering relieved by the boldness of modern surgery.

There is, however, a danger connected with this subject which is liable to be lost sight of in our admiration of the enormous strides in the direction referred to. The value of drugs, in the control of disease is apt to be overlooked, and the profession is led to rely too exclusively upon the scalpel, the drainage tube and the antiseptic solution, to the exclusion of remedies whose potency has been conclusively proven. It has become, to some extent, a fashion to deride therapeutic measures as slow and uncertain, and to vaunt a cynical skepticism in regard to the whole range of internal medication. Pathology and operative surgery have supplanted materia medica and therapeutics, and the removal of a diseased organ has become a greater *desideratum* than the restoration of its integrity by patient scientific treatment.

What is needed in the profession to-day to offset this tendency is the presence of men who will be content to investigate the properties of drugs, their physiological and therapeutic effects,—men who are in a position to avail themselves of ample clinical material to study diseases and their treatment by medicines, and who are willing to spend the necessary time and labor to collect statistics and compare results, in order to arrive at positive and unassailable conclusions. If the statistics of the treatment of dysentery or typhoid fever by drugs could be as industriously and as accurately collated as are the statistics of Cæsarian section or vaginal hysterectomy, there can be no doubt that results of incalculable value would follow such a study. An illustration has recently been furnished, in a partial investigation of the modern treatment of pneumonia, which has led to the startling conclusion that we are treating this disease much less successfully to-day than did our fathers fifty years ago. It cannot be that drugs have lost their power to control disease. The decadence lies in our appreciation of their power and in our knowledge of their proper use. If the general practitioner of to-day desires to emphasize his deprecation of the marked tendency to specialism, he can best accomplish this purpose by emulating the ardor, the enthusiasm and the untiring energy which characterize the work of the specialists.

The broad field of general practice presents many a pathway leading to distinction, and the distinction thus gained is more enviable than that which is offered to the specialists, just in proportion as the general diseases are of more frequent occurrence than abdominal tumors or cerebral abscesses. When Harley published his work on "The Old Vegetable Neurotics," he did a far greater service to the science of medicine than if he had reported any number of successful laparotomies. The older drugs of the Pharmacopœia have not yet been exhausted as fields for study. Even so homely and commonplace a medicine as Epsom salts has recently found its most valuable application in the control of a grave and often fatal condition, the acute peritonitis following abdominal section.

The general practitioner cannot afford to neglect the opportunities which are open to him. The law of supply and demand holds good here as in the mercantile world. Unless he shows himself able to successfully occupy his own legitimate domain, one specialty after another will be wrested from his grasp, until the little fragment which remains will be hardly worth the holding.

### ITEMS.

Dr. Carl Koller, who has achieved such world-wide renown in the discovery of the application of cocaine as a local anæsthetic, has been appointed Instructor in Ophthalmology at the New York Polyclinic.

THE AMERICAN ACADEMY OF MEDICINE.—The American Academy of Medicine is endeavoring to make as complete a list as possible of the Alumni of literary colleges in the United States and Canada who have received the degree of M. D. All recipients of both degrees, literary and medical, are requested to forward their names at once to Dr. R. J. Dunglison, Secretary, 814 N. 16th Street, Philadelphia, Pa.

"GLYCERINE AS A PURGATIVE."

Dr. Karl Ullmann (*Ctrlbl. f. die ges. Ther.*, August, 1888) contributes an exhaustive article on this subject, which already, in the short space of a year, can boast of quite an extensive literature. Mixed with water, glycerine enemata, he soon learned, were often attended with failure in chronic constipation. He employed a small glass syringe with its end covered with caoutchouc, and with a capacity of 3 ccm. (m. 45). A syringeful of pure glycerine was administered, and the cases selected were those in which constipation had existed for at least two or three days, the maximum duration being twelve days; 226 enemata altogether were given, and the result of each enema was noted separately. The quantity of the produced dejections varied; in 31.4 per cent. the stools were copious; in 45.5 per cent. they were moderate in amount; and in 23.1 per cent. they were very scanty, containing merely the injected glycerine, mixed with some mucus and a brownish fluid. In 83 per cent. the stools were hard, consisting chiefly of scybala; in 13 per cent. they were pappy and unformed; and in 4 per cent. they were a of diarrhœic nature. Subsequent constipation occurred in 39 per cent. Diarrhœa followed only in 3 per cent. Painful sensations during the stool, or immediately afterwards, such as a burning in the rectum and colic, were complained of only by five patients, three of whom were suffering from acute parametritis, one from retroversion of the uterus, and one from chronic tubercular peritonitis. Borborygmi existed in 4 per cent. only. Abnormal quantities of mucus occurred in four cases. Blood in quantities sufficient to be noticeable was observed in two cases. Of the fifty-two cases in which the enemata failed, the author could find no cause for the failure in the primary disease. In all, however, he was enabled to detect by palpation an atonic condition of the rectum, or an accumulation of the fæcal mass higher up than usual. The author favors the theory that the local purgative action of glycerine is due to its power of abstracting water from the tissues, and in this way irritating the rectal mucous membrane and causing contraction of the muscles of the rectum. Enemata of glycerine are serviceable where, for any reason, a rapid evacuation of the lower part of the bowel is indicated. They are of no use when the accumulation of fæces is in the upper part of the large bowel, or in the small intestines. They are contra-indicated in ulcerative conditions of the rectum, and in painful inflammatory affections of neighboring parts. In habitual constipation they cannot replace dietetic regimen, massage of the bowels, and well-known aperients. Further observations are necessary to determine whether their continued use is not injurious to the rectal mucous membrane.

### TREATMENT OF BALDNESS.

Besnier asserts that the falling out of the hair may be checked and a new growth started by the following treatment. The hair should be cut short, and a mild sinapism or rubefacient applied to the scalp, then every five days the following lotion is to be applied:—R. Acid acet., chloroformi āā q.s. The above should be used cautiously, as it is an irritant. The following pomade should be used with the lotion:—℞. Acidi salicyl. 15 grains, sulph. precip. 1½ drachms, vaseline 5 drachms. This pomade should be applied fresh every morning, the scalp having been previously washed. Fatty substances retard the growth of the hair, and should be avoided. (*Journ. de Med. de Paris.*)—*Brit. Journ. of Dermat.*, September, 1889.

### PROGRAMME OF THE SOUTHERN SURGICAL AND GYNACOLOGICAL ASSOCIATION.

NASHVILLE, NOV. 12, 13, 14.

### PAPERS TO BE READ.

The President's Annual Address....................HUNTER McGUIRE, M. D. Richmond, Va.

Report of Gynecological Work, with Especial Reference to Methods..... .............R. B. MAURY, M. D., Memphis, Tenn.

Direct Herniotomy, with Cases.....................W. O. ROBERTS, M. D., Louisville, Ky.

Open Abdominal Treatment........................B. E. HADRA, M. D., Galveston, Texas.

The Abortive Treatment of Acute Pelvic Inflammation....................................VIRGIL O. HARDON, M. D., Atlanta, Ga.

The Importance of Early Treatment of Inflammatory Affections of the Uterus.........WM. C. DABNEY, M. D., University of Virginia.

The Relation of the Nerve System to Reparative Surgery...............................THOS. O. SUMMERS, M. D., Jacksonville, Fla.

Concerning the Causes of Frequent Failure of Relief of Reflex Symptoms after Trachelorrhaphy...................................W. F. HYER, M. D., Meridian, Miss.

Cranial Surgery.......................................DESAUSSURE FORD, M. D., Augusta, Ga.

The Treatment of Ectopic Pregnancy.............W. H. WATHEN, M. D., 1 ouisville, Ky.

Laparotomy in Extra-Uterine Pregnancy........WALDO BRIGGS, M. D., St. Louis, Mo.

Epithelioma of the Penis, with the Report of Case ...............................................D. W. YANDELL, M. D., Louisville, Ky.

Laparotomy in Intestinal Obstruction............C. KOLLOCK, M.D., Cheraw, S. C.

An Experimental Study of Intestinal Anastomosis...............................................JOHN D. S. DAVIS, M. D., Birmingham, Ala.

Operative Interference in Ascites......... ........HUGH M. TAYLOR, M. D., Richmond, Va.

Observations pertaining to Pregnancy and Parturition..............................................W. DUNCAN, M. D., Savannah, Ga.

Puerperal Convulsions.................................J. HERBERT CLAIBORNE, M. D., Petersburg, Va.

Some Remarks upon Aneurisms, Relating More Especially to their Surgical Treatment .................. ...............................F. T. MERIWETHER, M. D., Asheville, N. C.

Coccygodynia and its Treatment....................HUNTER P. COOPER, M. D., Atlanta, Ga.

The Improved Cæsarean Section *versus* Craniotomy .................................................W. D. Haggard, M. D., Nashville, Tenn.

Conservative Surgery in Injuries of the Foot...J. T. WILSON, M. D.,
Sherman, Texas.

Gun-Shot Fracture of the Femur..................JOHN BROWNRIGG, M. D.,
Columbus, Miss

Tropho-Neurosis as a Factor in the Phenomena of Syphilis............... .......................G. FRANK LYDSTON, M. D.,
Chicago, Ill.

Trophic Changes Following Nerve Injury in Fractures, with a Report of Two Cases...WM. PERRIN NICOLSON, M. D.,
Atlanta, Ga.

Treatment of Malignant Diseases of the Rectum...... .........................................W. T. BRIGGS, M. D.,
Nashville, Tenn.

Gynecology in its Relation to Obstetrics.........W. L. ROBINSON, M. D.,
Danville, Va.

The Treatment of Urethral Stricture............F.W. MCRAE, M.D., Atlanta, Ga.

Observations Based upon an Experience of Seventy-five Abdominal Operations.........JOS. TABER JOHNSON, M. D.,
Washington, D. C.

Twenty Consecutive Cases of Abdominal Section.................................................L. S. MCMURTRY, M. D.,
Danville, Ky.

Triple Amputations....................................J. B. LUCKIE, M. D.,
Birmingham, Ala.

The Treatment of Contracted Bladder by Hot Water Dilatation.....................................I. S. STONE. M. D., Lincoln, Va.

Complications Occurring in the Clinical History of Ovarian Tumors........................RICHARD DOUGLAS, M. D.,
Nashville, Tenn.

What Kind of Instruments Does Modern Antiseptic Surgery Demand....................... J. W. LONG, M. D.,
Randleman, N. C.

Intestinal Anastomotic Operations with Segmented Rubber Rings, with Some Practical Suggestions as to their Use in other Surgical Procedures................................A. V. L. BROKAW, M. D.,
St. Louis, Mo.

Leucocythæmic Tumors as a Neoplastic Exponent of Rheumatism and their Similarity to Malignancy, with a Case...................W. LOCKE CHEW, M. D.,
Birmingham, Ala.

What Civilization is Doing for the Human Female ..................... ...........................A. LAPTHORN SMITH, M. D.,
Montreal, Canada.

The Achievements of Modern Surgery...........J. EWING MEARS, M. D.,
Philadelphia, Pa.

The Treatment of the Pedicle in Suprapubic Hysterectomy.........................................WM. M. POLK, M. D., New York

Pus in the Pelvis and How to Deal with It ....JOSEPH PRICE, M, D.,
Philadelphia, Pa.

The Railroads will sell tickets to those attending the meeting at reduced rates. Ask your Railroad Agent for return ticket.

HUNTER McGUIRE, M. D.,
*President.*

W. E. B DAVIS, M. D.,
*Secretary.*

